# Cohesin is required for expression of the estrogen receptor-alpha (*ESR1*) gene

**DOI:** 10.1186/1756-8935-5-13

**Published:** 2012-08-22

**Authors:** Tanja Prenzel, Frank Kramer, Upasana Bedi, Sankari Nagarajan, Tim Beissbarth, Steven A Johnsen

**Affiliations:** 1 Department of Molecular Oncology, Göttingen Center for Molecular Biosciences, University Medical Center Göttingen, Ernst-Caspari-Haus, Justus-von-Liebig-Weg 11, Göttingen 37077, Germany; 2 Department of Medical Statistics, University Medical Center Göttingen, Humboldt Allee 32, Göttingen, 37073, Germany; 3 Department of Tumor Biology, University Medical Center Hamburg-Eppendorf, Martinistraße 52, Hamburg 20246, Germany

**Keywords:** Estrogen receptor, Cohesin, Mediator, Chromatin

## Abstract

**Background:**

In conjunction with posttranslational chromatin modifications, proper arrangement of higher order chromatin structure appears to be important for controlling transcription in the nucleus. Recent genome-wide studies have shown that the Estrogen Receptor-alpha (ERα), encoded by the *ESR1* gene, nucleates tissue-specific long-range chromosomal interactions in collaboration with the cohesin complex. Furthermore, the Mediator complex not only regulates ERα activity, but also interacts with the cohesin complex to facilitate long-range chromosomal interactions. However, whether the cohesin and Mediator complexes function together to contribute to estrogen-regulated gene transcription remains unknown.

**Results:**

In this study we show that depletion of the cohesin subunit SMC3 or the Mediator subunit MED12 significantly impairs the ERα-regulated transcriptome. Surprisingly, SMC3 depletion appears to elicit this effect indirectly by rapidly decreasing *ESR1* transcription and ERα protein levels. Moreover, we provide evidence that both SMC3 and MED12 colocalize on the *ESR1* gene and are mutually required for their own occupancy as well as for RNAPII occupancy across the ESR1 gene. Finally, we show that extended proteasome inhibition decreases the mRNA expression of cohesin subunits which accompanies a decrease in *ESR1* mRNA and ERα protein levels as well as estrogen-regulated transcription.

**Conclusions:**

These results identify the *ESR1* gene as a cohesin/Mediator-dependent gene and indicate that this regulation may potentially be exploited for the treatment of estrogen-dependent breast cancer.

## Background

Transcriptional control is a highly ordered and complex process involving interactions between various transcription factors and the transcriptional apparatus [1]. Importantly, changes in chromatin organization, including post-translational histone modifications and higher order chromatin structure direct transcriptional activity and control gene expression patterns [2,3]. Recent studies have begun to uncover the complexity of interactions between different chromatin loci on a genome-wide level [4-6].

The estrogen receptor-alpha (ERα) is a ligand-activated transcription factor which plays an essential role in directing tissue-specific gene expression [7]. Importantly, ERα is a primary target for anti-estrogen therapy in breast cancer and its presence is a prognostic marker for patient outcome [7,8]. Interfering with distinct aspects of ERα-regulated transcription may provide novel therapeutic options for the treatment of ERα-positive breast cancer [9]. During gene transcription ERα functions not only to recruit an intricate network of transcriptional coregulators, but also nucleates long-range chromosomal interactions [4]. These functions are important for directing cell type-specific transcriptional programs [10].

Long-range chromosomal interactions appear to be stabilized by the cohesin complex [11]. In mammals, the cohesin complex consists of a ring structure containing SMC1A and SMC3 which is held together by the proteins RAD21 and STAG1/STAG2. Cohesin plays a central role in sister chromatid cohesion during and following DNA replication [11]. Consistently, mutations in the cohesin complex have been found in different types of cancer and have been linked to aneuploidy [12,13]. Interestingly, mutations in the Nipped-B-like (*NIPBL*) gene [14-16], which loads the cohesin complex onto chromatin, or mutations in *SMC1A* or *SMC3*[17-19] lead to a developmental phenotype called Cornelia de Lange Syndrome (CdLS). In addition to developmental phenotypes such as delayed neurodevelopment and other structural abnormalities, CdLS patients frequently exhibit potential endocrine-related defects such as slower pubertal growth and irregular menstrual cycles [20]. Consistent with a potential particular importance of cohesin in endocrine-regulated processes, cohesin subunits have been implicated in transcriptional regulation by some nuclear hormone receptors in both mammalian cells and in *Drosophila*[4,10,21-24]. Furthermore, a number of studies indicate that the phenotypes observed in CdLS patients are likely not due to defects in sister chromatid cohesion, but rather to changes in transcription [25,26].

Mediator is a large multisubunit complex which interacts directly with a number of transcription factors to facilitate RNA Polymerase II (RNAPII) recruitment to target genes [27]. Recent studies indicated that Mediator also interacts with cohesin to control long-range chromosomal interactions in embryonic stem cells [28]. Given the important role of Mediator in controlling ERα function [29-31] and the potential importance of cohesin in controlling ERα-nucleated long-range chromosomal interactions [4,10], we hypothesized that these complexes may similarly control estrogen-regulated gene expression. Our results show that depletion of components of either the cohesin or Mediator complexes significantly impairs ERα-regulated gene transcription. Surprisingly, this effect appears to be due to decreased transcription of the *ESR1* gene following SMC3 knockdown. These effects could be mimicked by prolonged treatment with the clinically utilized proteasome inhibitor bortezomib which also decreased the mRNA levels of *ESR1* and several cohesin subunits as well as SMC3 and ERα protein levels. These results provide an important molecular insight into the possible clinical benefit of proteasome inhibitor treatment for estrogen-dependent breast cancer and may also explain potential endocrine phenotypes frequently observed in patients with CdLS.

## Results

### SMC3 and MED12 knockdown decrease ERα-dependent transcription

**Figure 1:**
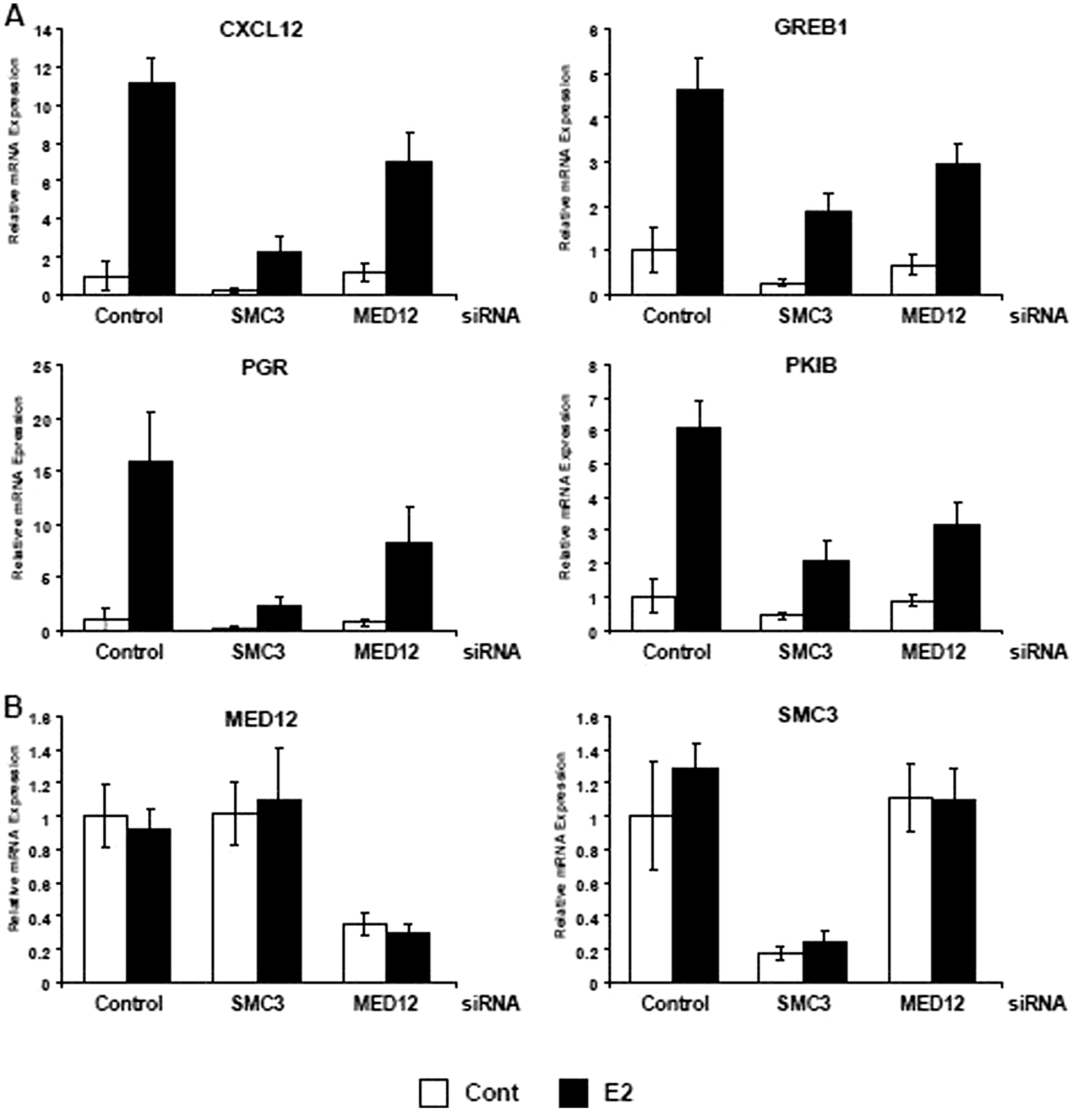
**SMC3 and MED12 depletion significantly reduces the estrogen-induced expression of ERα target genes.** MCF7 cells transfected with control, SMC3 or MED12 siRNA were grown in normal growth medium for 24 h and then for another 42 h in hormone-depleted medium before treating with 10 nM 17β-Estradiol (E2) for 6 h as indicated. Total mRNA was extracted, reverse-transcribed and analyzed by quantitative real-time PCR (qRT-PCR). (**A**) The expression levels of the estrogen-regulated genes *CXCL12*, *GREB1*, *PGR*, and *PKIB* were normalized to 28S ribosomal mRNA, graphed relative to the control sample and expressed as relative mRNA expression; mean values + SD, n = 4. (**B**) Efficient knockdown of *MED12* and *SMC3* was verified by qRT-PCR in the same samples used in (**A**). *MED12* and *SMC3* were normalized and expressed as in (**A**). Mean values + SD, n = 4.

In order to determine whether cohesin and Mediator are both required for estrogen-regulated transcription, we performed siRNA-mediated knockdown studies of both SMC3 and MED12 in MCF7 breast cancer cells. We investigated the expression of the *CXCL12*, *GREB1*, *PGR* and *PKIB* genes which we previously identified as robust estrogen-regulated genes in transcriptome-wide studies [32] and which all demonstrate occupancy of cohesin components and ER/cohesin-based chromosomal looping [4,10,32]. As shown in Figure 1A (and Figure 1A in Additional file 1), depletion of SMC3, MED12 or RAD21 significantly decreased the estrogen-induced mRNA levels of each of the estrogen-regulated genes tested. Notably, SMC3 knockdown also decreased the basal expression of these estrogen-regulated genes even in the absence of estrogen. These effects are reminiscent of those observed upon treatment with the pure anti-estrogen ICI 182,780, where both estrogen-induced and basal expression of estrogen responsive genes is affected by anti-estrogen treatment [33]. Thus, both basal and estrogen-induced expression of estrogen target genes appears to be dependent upon cohesin and Mediator for its activity. In contrast, *SMC3* and *MED12* mRNA expression was unaffected by either estrogen treatment or knockdown of the other component (Figure 1B).

**Figure 2:**
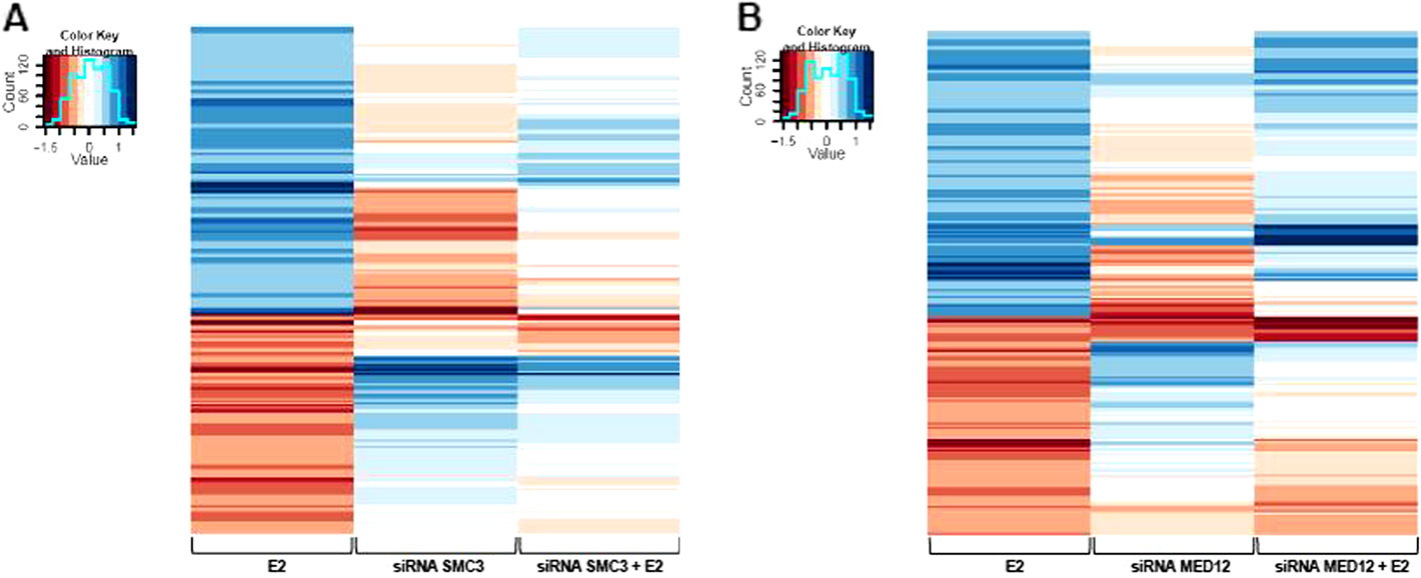
**SMC3 or MED12 depletion significantly impairs the estrogen-regulated transcriptome.** mRNA expression profiling of estrogen-regulated genes after *SMC3* (**A**) or *MED12* (**B**) depletion and 10 nM 17β-Estradiol (E2) treatment for 6 h. The heatmaps show log2-fold-changes in experiments control siRNA + 17β-Estradiol vs. control siRNA alone (E2), SMC3 (**A**) or MED12 (**B**) siRNAs vs. control siRNA (siRNA SMC3 or siRNA MED12) and SMC3 (**A**) or MED12 (B) siRNAs + 17β-Estradiol vs. control siRNA (siRNA SMC3 + E2 or siRNA MED12 + E2) (columns) for genes that are significantly (q < 0.05; q = *P*-values adjusted to False discovery rate regulated by estrogen (Fold-change, FC < −log2 (1.5) or FC > log2 (1.5)) (rows). The color keys range from red marking downregulated, to blue marking upregulated genes; mean values; n = 3.

To further examine the extent of the effects of SMC3 and MED12 knockdown on ERα activity, we performed transcriptome-wide analyses comparing the effects of each individual knockdown on the transcriptional profile of MCF7 cells both in the presence and absence of estrogen. As shown in Figure 2A, SMC3 depletion significantly blocked the overwhelming majority of estrogen-regulated gene transcription in MCF7 cells (compare Figure 2A left and right columns). Moreover, consistent with the single gene studies in Figure 1A, even the basal levels of expression were significantly affected (middle column). This effect was not limited to transcriptional activation since estrogen-repressed gene transcription was also reversed. A similar, albeit somewhat weaker, effect was also observed for MED12 knockdown (Figure 2B).

### Cohesin or mediator knockdown impairs *ESR1* gene expression

**Figure 3:**
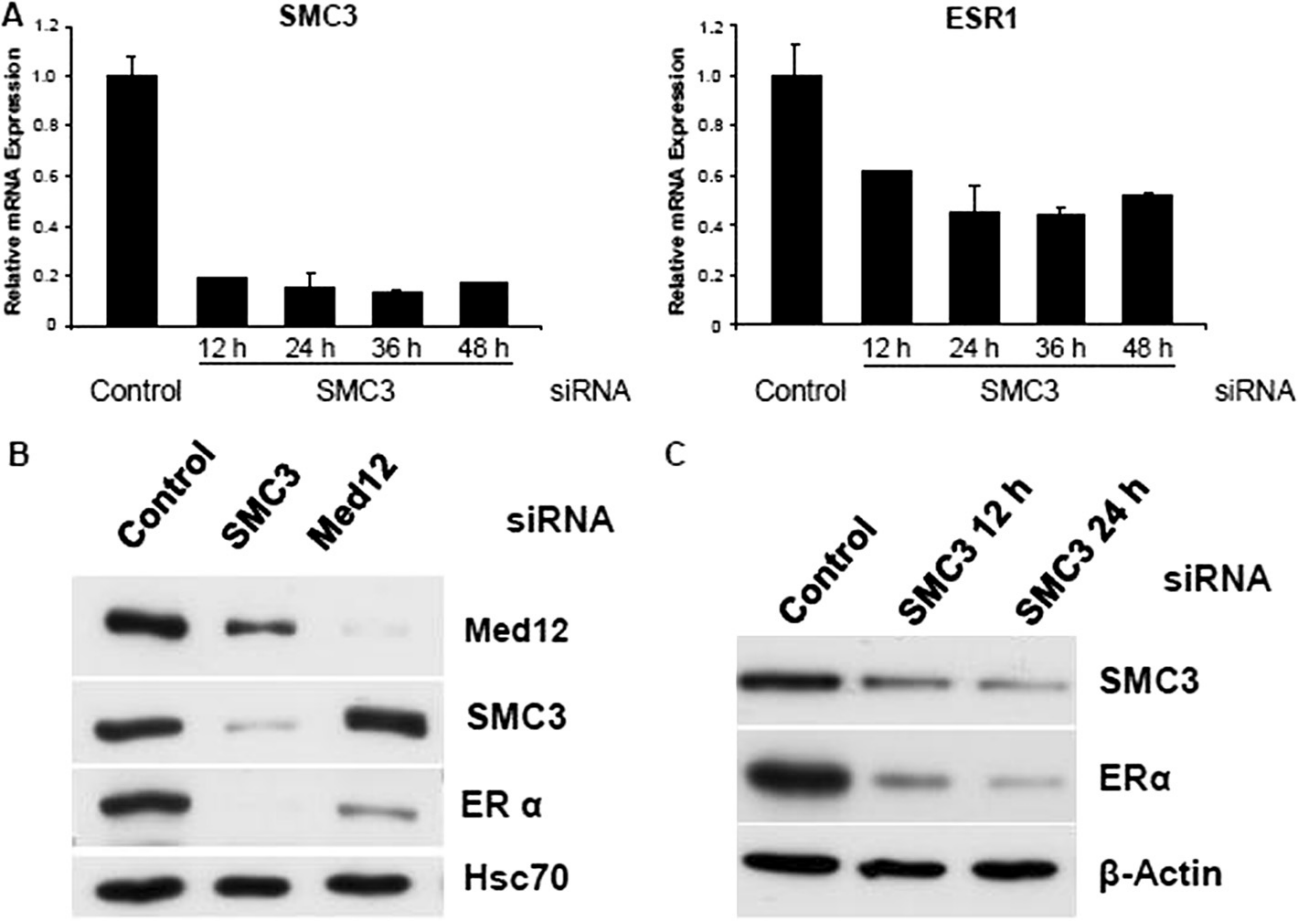
***SMC3 *knockdown decreases ERα gene expression and protein level.** (**A**) MCF7 cells were transfected with control or SMC3 siRNA. After cell adhesion, growth medium was changed to hormone-deprived 5% CSS medium. Total mRNA was harvested 12, 24, 36 and 48 h after transfection. *SMC3* and *ESR1* expression was normalized to 28S ribosomal mRNA, graphed relative to the control sample and expressed as relative mRNA expression; mean values + SD, n = 2. (**B**) MCF7 cells were transfected with control, SMC3 or MED12 siRNAs and grown overnight. Cells were switched to hormone-free medium for 32 hours before harvesting whole protein extracts and analysis by Western blot for MED12, SMC3, ERα and HSC70 protein levels. (**C**) MCF7 cells were transfected as in (**A**). Whole protein extracts were analyzed via Western blot with specific antibodies for SMC3 and ERα. β-Actin and HSC70 are shown as loading controls.

Unexpectedly, in the process of bioinformatic analysis of these transcriptome data, we found *ESR1* mRNA levels were also SMC3-dependent. We therefore performed time course analyses of RNA (Figure 3A) and protein (Figure 3B) expression to determine the kinetics of *SMC3* and *ESR1* regulation upon SMC3 depletion. These studies revealed a very rapid and parallel downregulation of *SMC3* and *ESR1* RNA levels already at 12 h after transfection of SMC3 siRNA (Figure 3A). Similarly, RAD21 knockdown also decreased *ESR1* mRNA levels as well (Figure 1B in Additional file 1). ERα protein levels were also decreased following knockdown of either SMC3 or MED12 (Figure 3B). Surprisingly, despite their reported long half-lives, both SMC3 and ERα protein levels were similarly decreased already 12 h post-transfection (Figure 3C). Thus, *ESR1* expression is highly dependent upon cohesin and Mediator.

### SMC3 and MED12 are required for mutual occupancy and transcription across the *ESR1* gene

**Figure 4:**
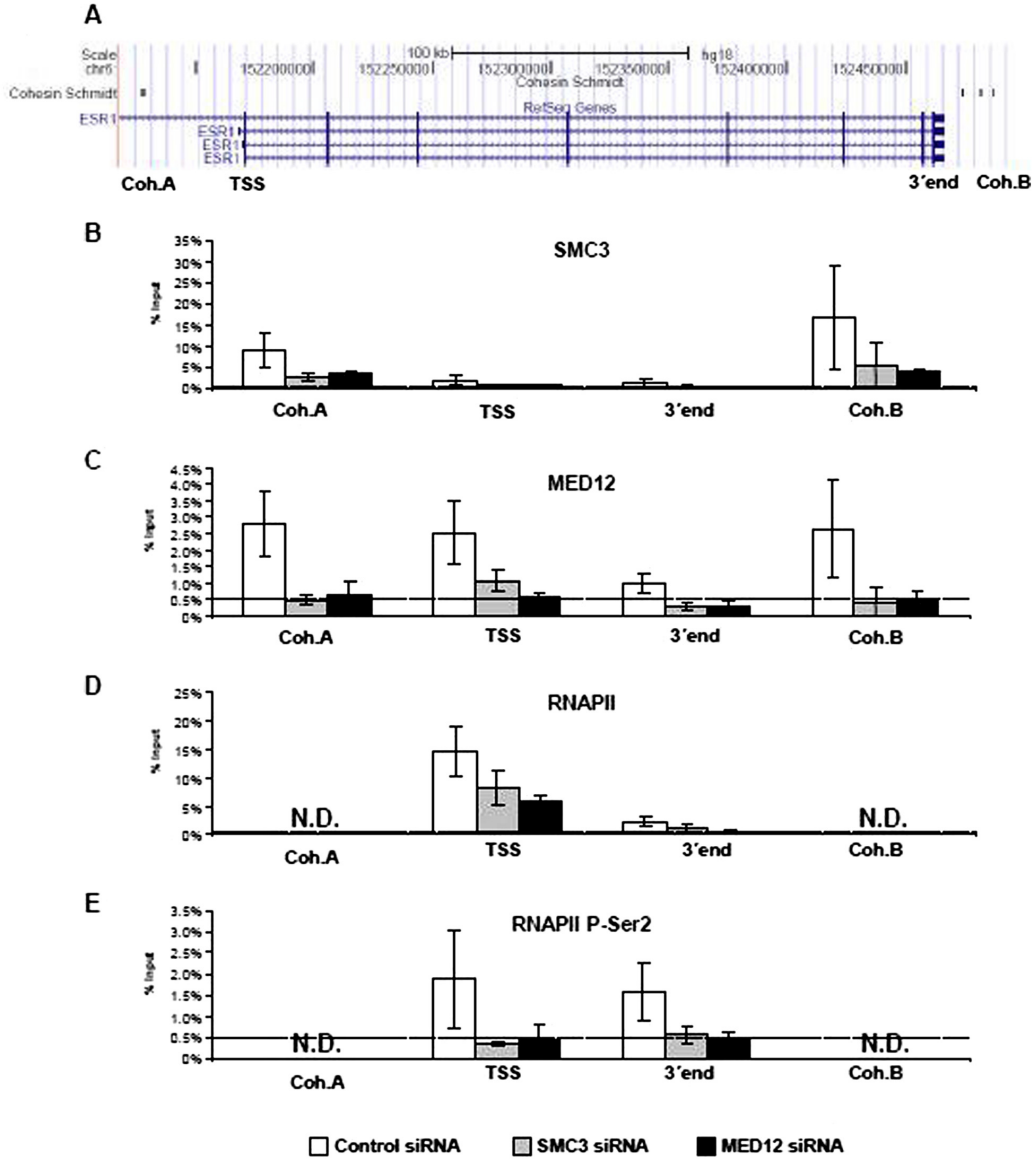
**The *ESR1 *gene is a target of regulation by the cohesin and Mediator complexes.** (**A**) A snapshot from the UCSC genome browser showing the structure of the *ESR1* gene and cohesin binding sites previously identified by chromatin immunoprecipitation-sequencing (ChIP-Seq) [10]. The indicated positions shown for the investigated cohesin binding sites as well as the transcriptional start site (TSS) and the 3^′^ end. (B-E) MCF7 cells transfected with control, SMC3 or MED12 siRNAs for 24 h were grown in estrogen-free medium for an additional 48 h. SMC3 (**B**), MED12 (**C**), RNAPII (**D**) and RNAPII P-Ser2 (**E**) binding to the transcriptional start site (TSS), two different cohesin binding sites (Coh. A and B), and the 3^′^ end (**A**) was analyzed using specific antibodies by chromatin immunoprecipitation analysis. ChIP samples were normalized to input samples and expressed as % input; mean values + SD, n = 3. The dotted line indicates the background binding as measured by the average signal of non-specific IgG binding across all samples and sites.

Previous chromatin immunoprecipitation-sequencing (ChIP-seq) studies revealed cohesin binding sites genome-wide in both the presence and absence of estrogen in MCF7 cells [10]. Analysis of these data revealed a number of cohesin binding sites on the *ESR1* gene (Figure 4A). We therefore performed ChIP analyses to verify cohesin binding to the *ESR1* gene. As shown in Figure 4B, SMC3 was present at both of the investigated cohesin binding sites, but at much lower levels near the transcriptional start site (TSS) or 3^′^ end of the *ESR1* gene. Importantly, SMC3 occupancy was decreased by either SMC3 or MED12 knockdown.

Consistent with a cooperative function with SMC3, MED12 was also present at both cohesin binding sites (Figure 4C). In addition, it was also found in significant levels at the TSS, but not at the 3^′^ end. This finding is consistent with the important role of the Mediator complex in transcriptional initiation.

To determine the effects of SMC3 and MED12 depletion on RNAPII-dependent *ESR1* transcription, we also investigated the occupancy of both total and elongating RNAPII (phosphorylated at Ser2 of the heptapeptide repeat in the C-terminal domain). Consistent with the decreased *ESR1* mRNA levels observed in Figure 3A, total RNAPII levels were decreased at the TSS and 3^′^ end of the *ESR1* gene after SMC3 or MED12 siRNA transfection (Figure 4D). However, significant levels of RNAPII were still present even after depletion of SMC3 or MED12 depletion. The effects of SMC3 or MED12 were more apparent when investigating the effects on elongating RNAPII. Surprisingly, while P-Ser2 RNAPII is generally enriched at higher levels in the transcribed region and 3^′^ end of active genes, significant levels were observed at both ends of the ESR1 gene. These results are similar to that observed for other genes, which demonstrated similar or higher levels of P-Ser2 RNAPII at the TSS compared to the transcribed region [34]. Importantly, the levels of P-Ser2 RNAPII were reduced to background levels at both the TSS and 3^′^ end in the absence of either SMC3 or MED12 (Figure 4E). Thus, the knockdown of components of either the cohesin or Mediator complexes may affect both transcriptional initiation as well as elongation.

### Bortezomib decreases *SMC3* and *ESR1* gene expression

**Figure 5:**
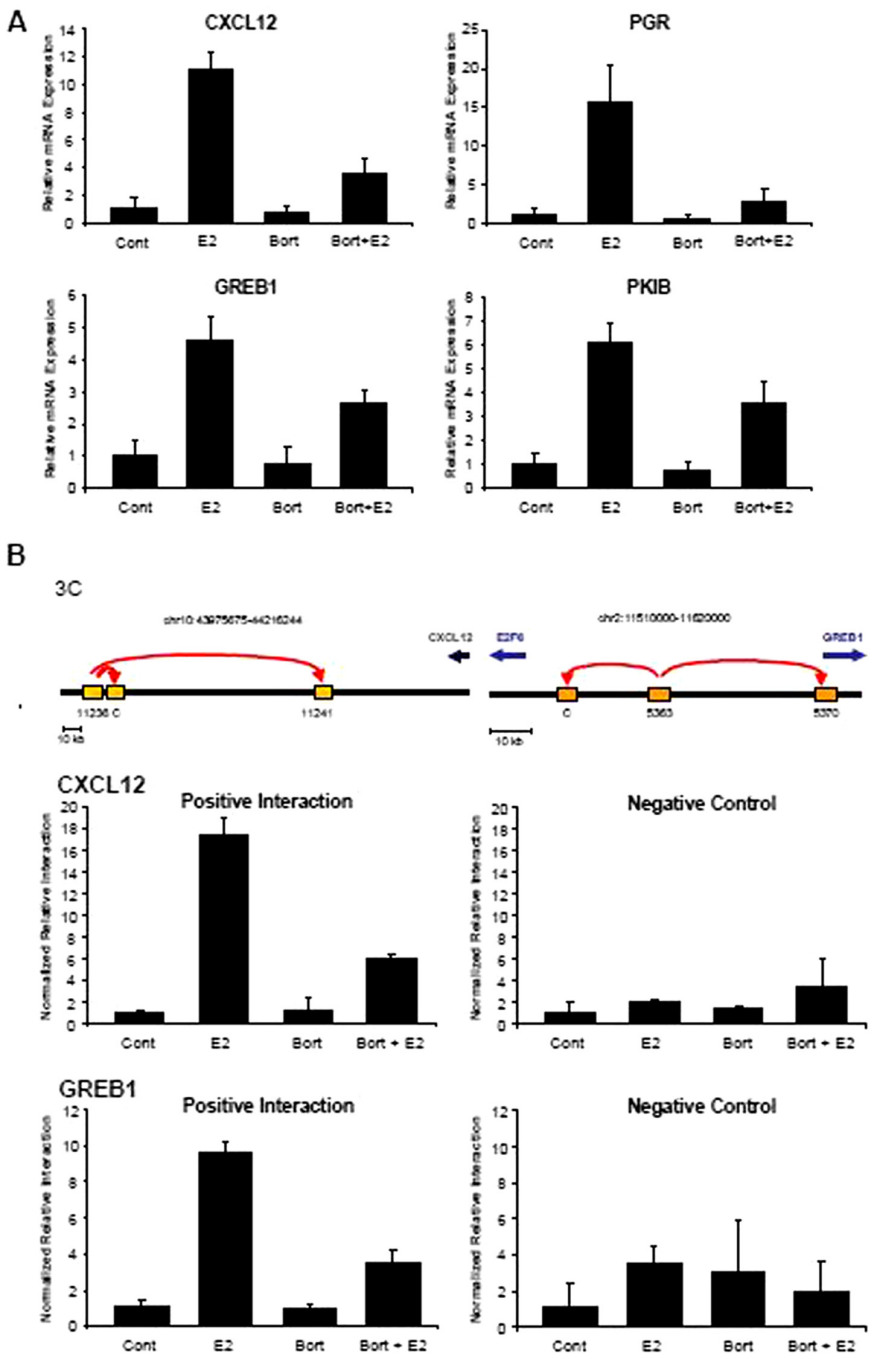
**Proteasome inhibition using bortezomib negatively influences estrogen-induced gene regulation.** (**A**) After pre-treatment with 50 nM bortezomib (Bort) or vehicle (ethanol, Cont) for 15 minutes, MCF7 cells were incubated with 10 nM 17β-Estradiol (E2) for 6 h before extracting total mRNA. The expression levels of estrogen target genes *CXCL12*, *GREB1*, *PGR* and *PKIB* were normalized to 28S ribosomal mRNA, graphed relative to the control sample and expressed as relative mRNA expression; mean values + SD, n = 4. (**B**) Chromatin conformation capture (3C) analysis of positive and negative interactions at the *CXCL12* and *GREB1* loci as identified by Schmidt *et al.*[10] and previously described [32] (upper panel). MCF7 cells, pre-treated with 50 nM bortezomib (Bort) or vehicle (ethanol, Cont) for 15 minutes, were incubated with 10 nM 17β-Estradiol (E2) for 24 h. Purified DNA samples were quantified by qPCR using a standard curve containing the respective BAC clones for *CXCL12* or *GREB1*. The 3C template values were normalized to values from an internal control site that lies between restriction enzyme sites, graphed relative to the control sample (set to 1) and represented as normalized relative interaction; mean values + SD, n = 3.

In a previous study we showed that short-term (2 h) treatment with the clinically utilized proteasome inhibitor bortezomib had no effect on ERα recruitment to target gene expression [32]. However, another study demonstrated that chronic treatment with bortezomib decreased *ESR1* mRNA and ERα protein levels [35]. Therefore, we also tested the effects of bortezomib treatment on estrogen-regulated gene expression in this system. Indeed, bortezomib treatment for 6 h significantly decreased the estrogen-induced mRNA levels of *CXCL12*, *PGR*, *GREB1* and *PKIB* (Figure 5A). These results paralleled a decrease in estrogen-induced long-range chromosomal interactions on both the *CXCL12* and *GREB1* genes (Figure 5B).

**Figure 6:**
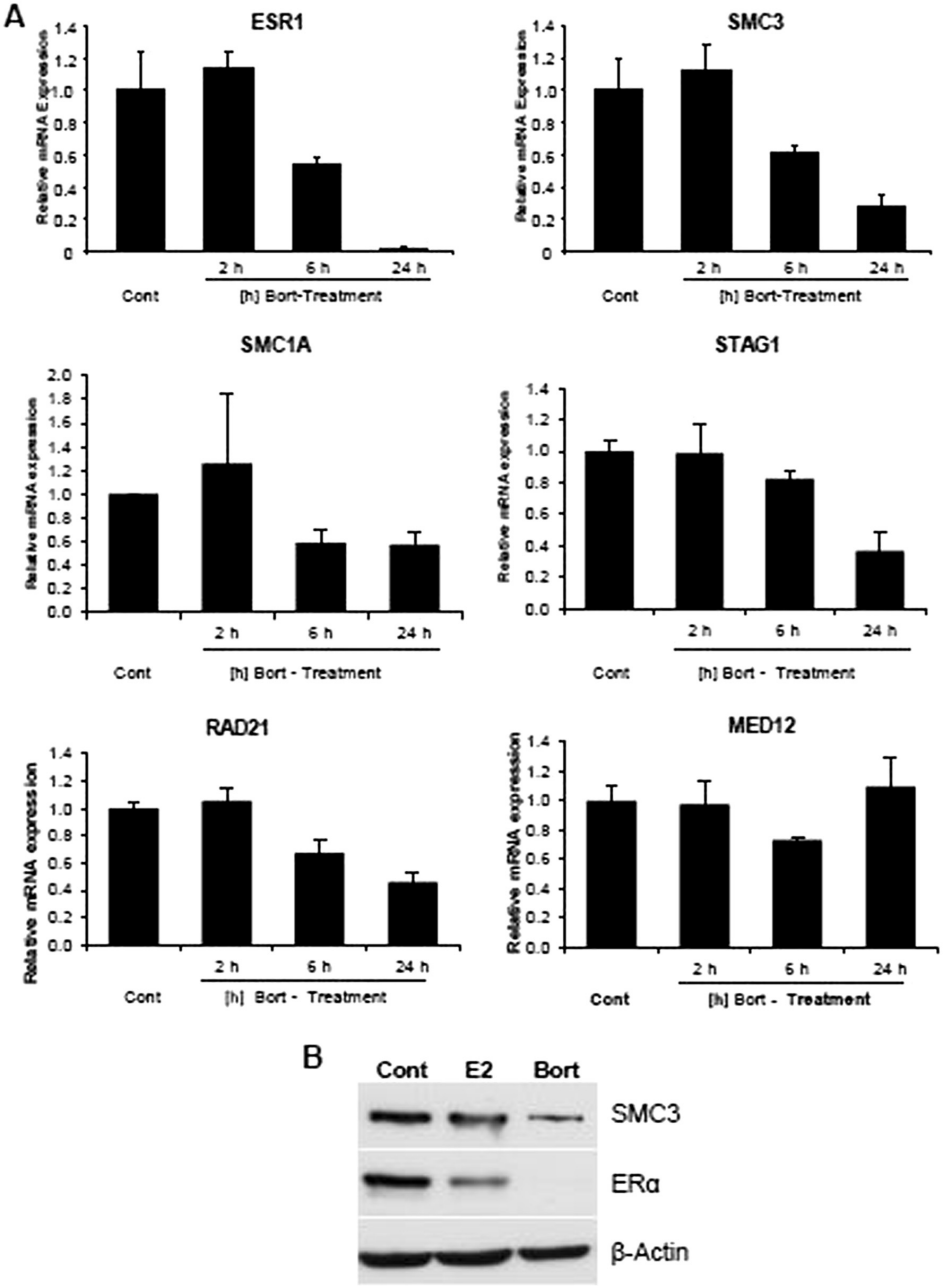
**Bortezomib treatment decreases ERα and cohesin expression.** (**A**) MCF7 cells were treated with vehicle (ethanol, Cont) or 50 nM bortezomib (Bort) for 2, 6 or 24 h. Total mRNA was harvested and reverse-transcribed. The expression levels of *ESR1, SMC3, SMC1A, STAG1, RAD21* and *MED12* genes were normalized to 28S ribosomal mRNA and graphed as in (**A**); mean values + SD, n =2. (**B**) MCF7 cells were treated with either vehicle (ethanol, Cont), 10 nM 17β-Estradiol (E2) or 50 nM bortezomib (Bort) for 24 h. SMC3 and ERα protein levels were analyzed by Western blot analysis. β-Actin is shown as loading control.

To verify the effects of bortezomib treatment on *ESR1* expression and to test whether a similar effect could be observed for cohesin and/or Mediator subunits, we examined *ESR1*, *MED12* and cohesin subunit mRNA levels at various time points following bortezomib treatment. As shown in Figure 6A, *ESR1* and *SMC3* showed similar profiles with the mRNA levels of both being unaffected at 2 h and greater effects at 6 and 24 h after treatment. Similar effects were also observed for *STAG1* and *RAD21*, and to a lesser extent *SMC1A*, whose mRNA levels also decreased following extended bortezomib treatment, albeit to differing degrees. In contrast, *MED12* mRNA levels were only mildly and transiently affected by bortezomib treatment. The effects on cohesin and ERα were also substantiated at the protein levels where bortezomib treatment resulted in decreased SMC3 and ERα protein levels (Figure 6B). In contrast, as previously reported [36], estrogen treatment alone also decreased ERα protein levels without affecting SMC3 levels.

### *ESR1* and *SMC3* regulation is not due to a cell cycle arrest

**Figure 7:**
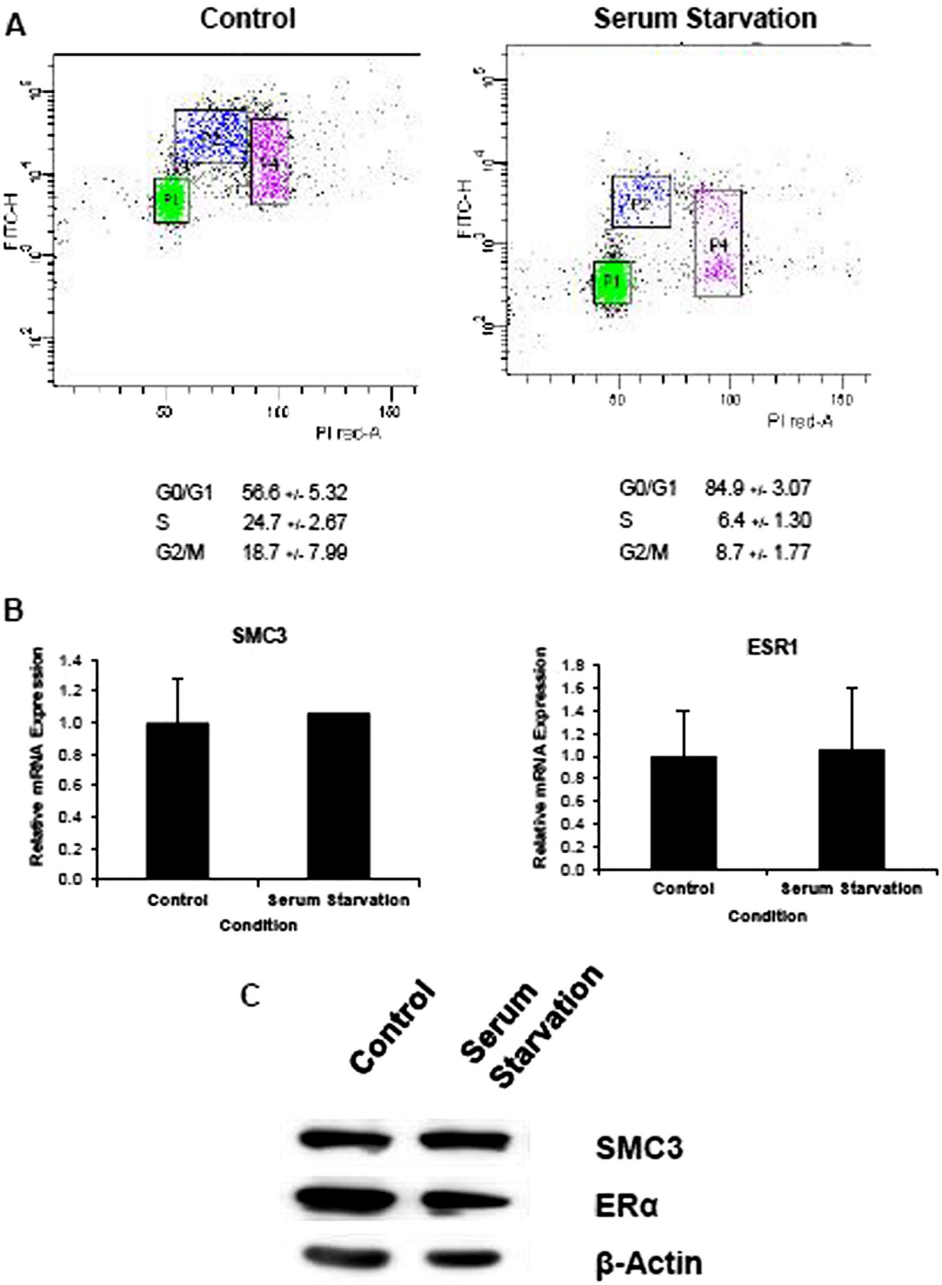
**Cell cycle arrest does not affect SMC3 and ERα expression.** (**A**) MCF7 cells were grown under normal growth or serum-free conditions and analyzed by bromodeoxyuridine (BrdU) and propidium iodide-based flow cytometry. Serum withdrawal induces a clear decrease in the S phase fraction and increase in the G1 cell fraction. (**B**) Total RNA from cells as treated in (A) was harvested, reverse-transcribed and gene expression was measured by qRT-PCR. *SMC3* and *ESR1* expression were normalized to 28S ribosomal mRNA, graphed relative to the control sample and expressed as relative mRNA expression; mean values + SD, n = 2. (**C**) MCF7 cells were grown as in (**A**) and (**B**) and SMC3 and ERα protein levels were analyzed by Western blot analysis. β-Actin is shown as loading control.

Given its essential role in cell cycle progression, it was conceivable that the changes observed in *SMC3* (and consequently also *ESR1*) gene expression upon bortezomib treatment (or cohesin knockdown) may be due to a general effect caused by a cell cycle arrest. Interestingly, while cohesin activity is required for sister chromatin cohesion, knockdown of either SMC3 or MED12 and further growth under hormone-free conditions resulted in a slight increase in the G1 fraction of cells compared to control transfected cells (Figure 2 in Additional file 1). In order to test whether the gene expression effects we observed were due to a G1 cell cycle arrest, we induced a G1 arrest by growing cells under serum-free conditions. Although this treatment substantially increased the G1 fraction of cells (Figure 7A), serum withdrawal had no effect on either *SMC3* or *ESR1* mRNA or protein levels (Figure 7B and C).

## Discussion

Genome organization within the nucleus is emerging as one of the most exciting, but highly complex gene expression regulatory mechanisms. The recent development of new laboratory techniques and the advent of next generation sequencing technologies have made it possible to analyze intra- and inter-chromosomal interactions on a genome-wide level. Despite these advances much still remains to be learned about the extensive network of proteins controlling genome organization and the plasticity of its regulation.

In this study we sought to determine the role of the cohesin and Mediator complexes in controlling estrogen-dependent gene transcription. While we saw a substantial reversal in the estrogen-regulated gene expression pattern following depletion of components of each complex, these effects may be an indirect effect caused by decreased *ESR1* expression. Consistently, both SMC3 and MED12 protein binding was detected on the *ESR1* gene and depletion of either component not only affected their occupancy, but also RNAPII occupancy on the *ESR1* gene. Thus, these data imply that a specific Mediator/cohesin-directed higher order chromatin structure is essential for proper *ESR1* expression. In the course of this study a number of possible interactions between various cohesin binding sites on the *ESR1* gene were tested by chromatin conformation capture analyses. Unfortunately, interactions between the investigated sites could not be observed (data not shown). This finding is consistent with recent data demonstrating that many long-range chromosomal interactions do not necessarily occur between adjacent cohesin binding sites but rather often encompass chromosomal loops of several hundred kilobases [4]. Thus it is currently impossible to precisely predict which cohesin binding sites and which chromatin interactions are essential for directing proper *ESR1* gene transcription. Additional studies in MCF7 cells using cohesin components for genome-wide chromosomal interaction analyses, similar to those recently reported for CTCF in embryonic stem cells [5], may help to uncover these interactions. Furthermore, additional non-looping functions of cohesin (for example, through direct interaction with other transcriptional regulatory proteins) cannot be excluded and must be considered.

Previous studies indicated a critical role for cohesin components in controlling ERα-regulated gene transcription [4,10]. Although cohesin likely plays a direct role in ERα-directed transcription, the finding that the expression of the *ESR1* gene itself is directly controlled by the cohesin complex suggests that caution will also need to be used when interpreting other siRNA-mediated knockdown studies of cohesin complex components. Similar effects were also recently observed when investigating the effects of cohesin on ecdysone receptor-regulated transcription [23,24]. One potential aspect which may affect the results in many studies is the cell cycle-dependent effects of cohesin knockdown. Given the important role of cohesin in sister chromatid cohesion, a knockdown of cohesin complex components will affect cell cycle progression. As previously reported [10] and demonstrated in our results, given the cell cycle arrest induced by estrogen withdrawal, this aspect likely does not influence the interpretation of our data. However, the molecular mechanism leading to the reported defect in estrogen-induced S-phase entry following depletion of the cohesin complex component RAD21 [10] may need to be reassessed. In this case, the effects observed may not be due to defects in the induction of ERα-directed chromosomal interactions, but rather due to an upstream loss of *ESR1* gene expression. Given the complexity of the role of different chromosomal interactions and the dramatic changes in transcriptional regulation that occur following cohesin complex component depletion, a number of secondary effects such as those observed in our study may occur. Furthermore, depletion of the cohesin complex will likely result not only in the loss of chromosomal loops that promote gene transcription (by bringing enhancers close to genes), but also loops which repress gene transcription (for example, cohesin binding sites which serve as insulators). The net effect of cohesin depletion will be a complex mixture of all of these effects.

One important aspect of these findings is their potential implication for the treatment of ERα-positive breast cancer. We confirmed the findings of another study [35] which clearly demonstrated that chronic bortezomib treatment decreases both *ESR1* mRNA and ERα protein levels. Although the effects of proteasome inhibition are highly pleiotropic, the finding that bortezomib treatment leads to a rapid and parallel decrease in both *SMC3* and *ESR1* gene expression opens an interesting new possible mechanism for its potential utility in the treatment of ERα-positive breast cancer. Although studies using bortezomib as a single agent to treat breast cancer were disappointing [37], new clinical studies are currently underway in which bortezomib is being tested in combination with a pure anti-estrogen specifically in ERα-positive metastatic breast cancer (NCT01142401). Given the results presented here, it would be particularly interesting to investigate ERα expression in patients receiving bortezomib treatment to determine if this effect is also observed *in vivo*. Given the potential role of cohesin downstream of the androgen receptor [21,22], combined anti-androgen and bortezomib treatment may also prove to be effective in combatting androgen-dependent prostate cancer.

Analogous to the effects of bortezomib treatment, other therapies may work by a similar epigenomic mechanism to decrease *ESR1* expression. For example, treatment of MCF7 cells with several different histone deacetylase inhibitors (HDACi) also resulted in decreased *ESR1* expression and a concomitant decrease in estrogen-regulated transcription [38]. Interestingly, although it is currently unknown whether HDACi treatment affects *SMC3* expression, the proteasomal subunit *PSMB2* is downregulated following HDAC inhibition [38]. Given the similar effects observed between proteasome inhibition and proteasome subunit knockdown [32], it is possible that the effects of HDACi treatment on *ESR1* expression may also occur as a secondary effect through downregulation of *SMC3* expression. Future studies will need to address whether the decrease in ERα levels following proteasome or HDAC inhibition has a positive or negative effect on tumorigenesis. While decreased *ESR1* expression may initially decrease tumor growth, it is possible that these treatments may, in fact, enhance the formation of more aggressive ERα-negative tumors.

Finally, the finding that *ESR1* expression is strongly dependent upon the integrity of the cohesin complex may also provide an insight into the molecular mechanisms behind various developmental phenotypes observed in CdLS. The question of whether *NIPBL*, *SMC1A* or *SMC3* mutation lead to decreased ERα levels in CdLS patients will need to be further addressed. Similarly, whether proteasome or HDAC inhibition lead to decreased ERα expression in patients, and how this leads to various side effects (that is, related to fertility) should also be investigated.

## Conclusion

Based on the results presented here, we propose that the *ESR1* gene is an important transcriptional target of cohesin and Mediator which is particularly sensitive to changes in the integrity of these complexes. It also uncovers a molecular mechanism and potential clinical utility for various therapies which alter *ESR1* expression as a secondary effect following changes in *SMC3* expression. Additional clinical studies, as well as cell culture and mouse model studies, will help to determine the clinical efficacy of these therapeutic strategies and their efficacy in the treatment of breast cancer.

## Methods

### Cell culture and RNA interference

MCF7 cells were obtained from the Department of Tumor Biology at the University Medical Center Hamburg-Eppendorf (Hamburg, Germany) and were grown in phenol red–free high-glucose DMEM (Invitrogen; Carlsbad, California, USA) supplemented with 10% bovine growth serum (Thermo Scientific; Waltham, MA, USA). MCF7 cells were grown in DMEM containing 5% charcoal-dextran–treated FBS (CSS; HyClone : Thermo Scientific, MA, USA) 1 to 2 days prior to treatment with 10 nmol/L 17-β-estradiol (Sigma-Aldrich; Missouri, USA) as indicated. Where indicated, cells were treated with 50 nmol/L bortezomib (LC Laboratories; Woburn, USA). Small interfering RNAs for SMC3 (Dharmacon; Thermo Scientific, USA; M-006834-01-0005), MED12 (Ambion; Invitrogen, USA; a pool of s19362, s19363 and s19364), or a negative control siRNA (Dharmacon; D-001206-13) were transfected using Lipofectamine RNAiMAX (Invitrogen) according to the manufacturer’s instructions. Flow cytometry experiments were performed as previously described [39].

### Antibodies and western blot

Antibodies for western blot and/or ChIP analyses were beta-actin (Abcam; Cambridge, UK; ab6278), ERα (Santa Cruz; CA, USA sc-543), non-specific IgG (Abcam; ab46540), MED12 (Bethyl; TX, USA ; ICH-00180), RNAPII (sc-899), Ser2-phosphorylated RNAPII CTD [40], and SMC3 (Abcam; ab9263). For Western blot analyses, cells were lysed in RIPA buffer containing 1 mM Pefabloc and 1 ng/μl aprotinin/leupeptin, separated by SDS-PAGE and transferred to a nitrocellulose membrane. Proteins were detected using the respective antibodies by enhanced chemiluminescence.

### Microarray and gene expression analysis

Total RNA was isolated from cells using the Qiazol reagent (Qiagen; Hilden, Germany) according to the manufacturer’s instructions. One microgram of total RNA was reverse transcribed using random nonamers (Metabion; Martinsried, Germany). Real-time PCR analysis was performed as previously described [32] using the following primers: *CXCL12*[32] (forward: 5^′^-TGCCAGAGCCAACGTCAAGCATC-3^′^; reverse: 5^′^-CGGGTCAATGCACACTTGTCTGTTGT-3^′^), *ESR1* (forward: 5^′^-GCATTCTACAGGCCAAATTCA-3^′^; reverse: 5^′^-TCCTTGGCAGATTCCATAGC-3^′^), *GREB1*[34] (forward: 5^′^-GTGGTAGCCGAGTGGACAAT-3^′^; reverse: 5^′^-ATTTGTTTCCAGCCCTCCTT-3^′^), 28S rRNA (forward: 5^′^-CTTTAAATGGGTAAGAAGCC-3^′^[32] ; reverse: 5^′^-ATCAACCAACACCTTTTCTG-3^′^), *MED12* (forward: 5^′^-ACAGGCTCCCATGCTGACGGA-3^′^; reverse: 5^′^-AAGGCAAGGTCCCCTCGGGAG-3^′^), PGR [32] (forward: 5^′^-TCCACCCCGGTCGCTGTAGG-3^′^; reverse: 5^′^-TAGAGCGGGCGGCTGGAAGT-3^′^), *PKIB*[32] (forward: 5^′^-ACGTGGAGTCTGGGGTCGCC-3^′^; reverse: 5^′^-GAGAGCCTCCAGTTTGAGGGGCA-3^′^), and *SMC3* (forward: 5^′^-GTTTCAACCCAGCTGGCCCGTG-3^′^; reverse: 5^′^-CGATGGCTGACTTGGTCACCTTCCA-3^′^). Gene expression was normalized to a control gene (28S rRNA) and expressed as fold induction relative to the untransfected, control condition. For whole transcriptome analyses, microarray studies were performed by the Vancouver Prostate Centre Laboratory for Advanced Genome Analysis (Vancouver, Canada) using the Illumina human HT-12 v4 beadchip. Gene expression data were analyzed as previously described (Prenzel *et al*., 2011). Heatmaps indicate genes which were at least 1.5-fold up- or downregulated with *P*-values ≤ 0.05. All gene expression data has been deposited into the GEO repository [accession number GSE38252].

### Chromatin immunoprecipitation and chromosome conformation capture assays

Chromatin immunoprecipitation was performed and analyzed by quantitative real-time PCR as previously described [32] using the following primers for various positions along the *ESR1* gene: TSS (forward: 5^′^-AAGTTGGAGGCCCGGGAGCC-3^′^; reverse: 5^′^-CCCGACGGGAGCAAGTGCAG-3^′^), cohesin binding site 1 (forward: 5^′^-CACGCTGGCTACATTTCAAGTGCTTCA-3^′^; reverse: 5^′^-AGTGCTGCCATCTACAGGGTCGAC-3^′^), cohesin binding site 2 (forward: 5^′^-AAACAGGCAGCACGCAGTGTTTCT-3^′^; reverse: 5^′^-GTGATTGAGCTCTTGTGGCTTCTTGGG-3^′^), 3^′^ end (forward: 5^′^-CCCCAGAGGCCGAGTGCCA-3^′^; reverse: 5^′^-CCTGCCTGGAAAGGTGACATGTGTG-3^′^). ChIP DNA samples were normalized to input DNA from the same sample and expressed as percent input. Chromatin conformation capture studies were performed as described previously [32] using the following primer/probe combinations: *CXCL12* positive interaction (forward: 5^′^-GAAGGAAGAAGAAACATGGACTCTGCTCCA-3^′^; reverse: 5^′^-ACAGAAGCTGGTTTACCGACTTGTCTGT-3^′^; internal probe: 5^′^-Fam-GCCCCAGGGCACAACACACC-BHQ1-3^′^), *CXCL12* negative control (forward: 5^′^-GAAGGAAGAAGAAACATGGACTCTGCTCCA-3^′^; reverse: 5^′^-CTCCCAGTGCAGAGGGAAGCATGT-3^′^; internal probe: 5^′^-Fam-GCCCCAGGGCACAACACACC-BHQ1-3^′^), *GREB1* positive interaction (forward: 5^′^-CTGGGCCTCTCCAGGGGGTTTT-3^′^; reverse: 5^′^- CCGCTGGTCAGCCGTTCAGG −3^′^; internal probe: 5^′^-Fam-GTCAGGGCAAAGGACATGGCCAG-BHQ1-3^′^), *GREB1* negative control (forward: 5^′^-GCCACTACATCCTTGGCTTTGTCCAC-3^′^; reverse: 5^′^- CCGCTGGTCAGCCGTTCAGG-3^′^; internal probe: 5^′^-Fam- GTCAGGGCAAAGGACATGGCCAG-BHQ1-3^′^), normalization control (within the GREB1 gene) (forward: 5^′^-GGGCTGGGTGCCCGTTTTGT-3’; reverse: 5^′^-CCAGCAGCTGCACGCCACAT-3’; internal probe: 5^′^-Fam-CCTGTGACATCTCTCCCAGCCCC-BHQ1-3’). All interactions were quantified by real-time PCR using standard curves containing digested and re-ligated DNA from BAC clones (ImaGenes; Berlin, Germany) covering the investigated regions of the *CXCL12* (RP-13309I17) and *GREB1* (RPCIB753E0150Q) genes and normalized using a probe-primer pair detecting an amplicon between BtgI sites as described previously [32].

## Abbreviations

BrdU: Bromodeoxyuridine; CdLS: Cornelia de Lange Syndrome; ChIP: Chromatin immunoprecipitation; CTD: C-terminal domain; ERα: Estrogen receptor-α; ESR1: Estrogen receptor 1; FC: Fold change; FDR: False discovery rate; HDACi: Histone deacetylase inhibitor; MED12: Mediator complex subunit 12; qRT-PCR: Quantitative real-time reverse transcription polymerase chain reaction; RNAPII: RNA Polymerase II; siRNA: Small interfering ribonucleic acid; SMC: Structural maintenance of chromosomes 3; TSS: Transcriptional start site.

## Misc

Frank Kramer and Upasana Bedi contributed equally to this work.

## Competing interests

The authors declare that they have no competing interests.

## Authors’ contributions

TP and SAJ designed the experiments. TP, UB and SN performed the experiments. FK and TB performed bioinformatic analyses of the microarray data. SJ wrote the manuscript. All authors read and approve the final manuscript.

## Supplementary Material

Additional file 1 **Figure S1.** RAD21 depletion significantly reduces the estrogen-induced expression of ERα target genes. MCF7 cells transfected with control or RAD21 siRNA were grown in normal growth medium for 24 h and then for another 42 h in hormone-depleted medium before treating with 10 nM 17β-Estradiol (E2) for 6 h as indicated. Total mRNA was extracted, reverse-transcribed and analyzed by quantitative real-time PCR (qRT-PCR). (**A**) The expression levels of the estrogen-regulated genes *CXCL12*, *GREB1*, *PGR*, and *PKIB* were normalized to 28S ribosomal mRNA, graphed relative to the control sample and expressed as relative mRNA expression; mean values + SD, n = 2. (**B**) Efficient knockdown of *RAD21* and its effects on *ESR1* mRNA levels was verified by qRT-PCR in the same samples used in (**A**). *RAD21* and *ESR1* were normalized and expressed as in (**A**). Mean values + SD, n = 2. **Figure S2.** SMC3 or MED12 knockdown enhance hormone-withdrawal induced G1 cell cycle arrest. MCF7 cells transfected with control, SMC3 or MED12 siRNA were grown in normal growth medium for 24 h and then for another 42 h in hormone-depleted medium before flow cytometric analyses. Shown are representative profiles and the respective quantitation for duplicate samples (± SD). Click here for file
