# Supplementary figures and images for: Cohesin is required for expression of the estrogen receptor-alpha (ESR1) gene
Source: Epigenetics Chromatin. 2012 Aug 22;5:13. doi: 10.1186/1756-8935-5-13 (PMC3488477; doi:10.1186/1756-8935-5-13)

Supplementary Figure 1

A

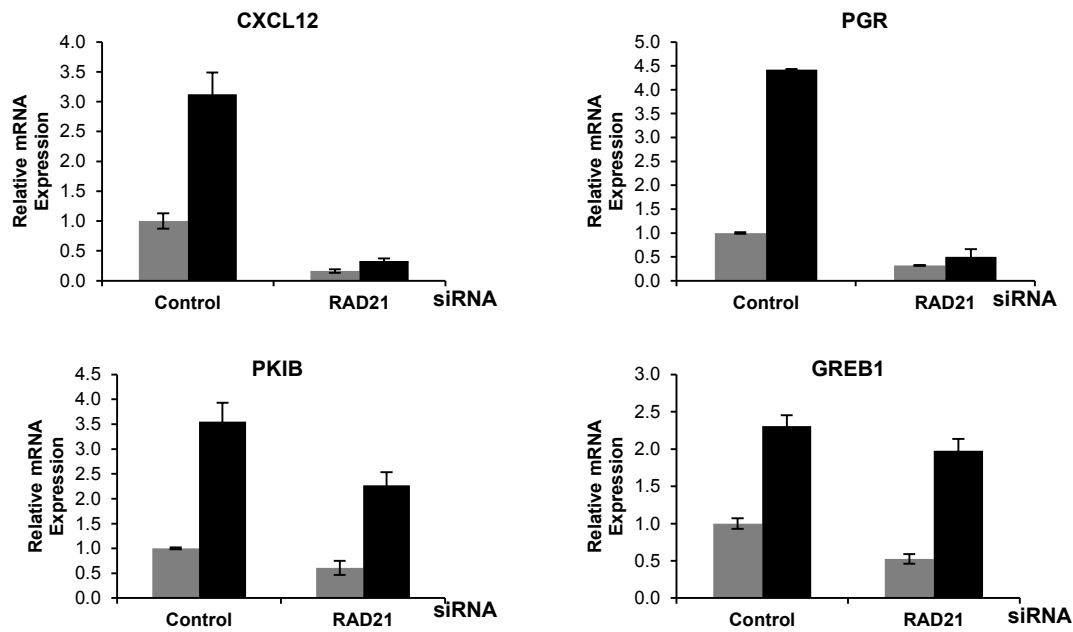

B

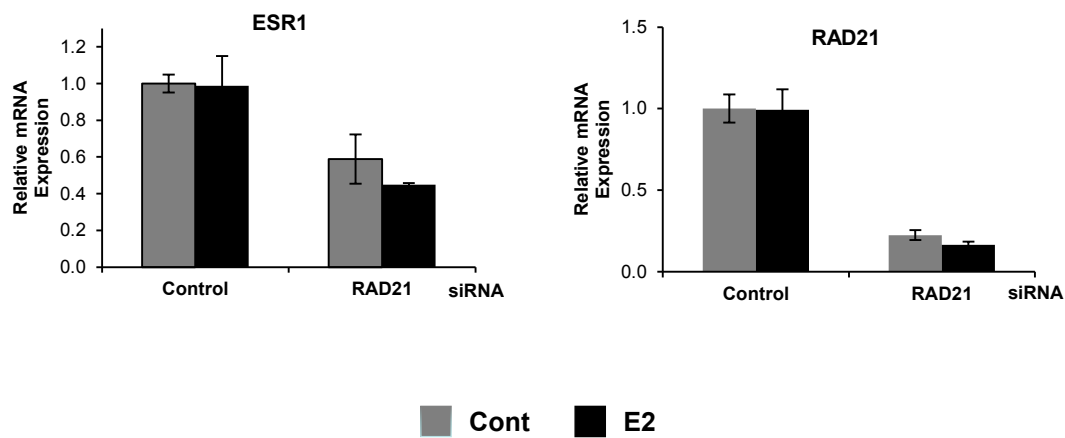

Supplementary Figure 2

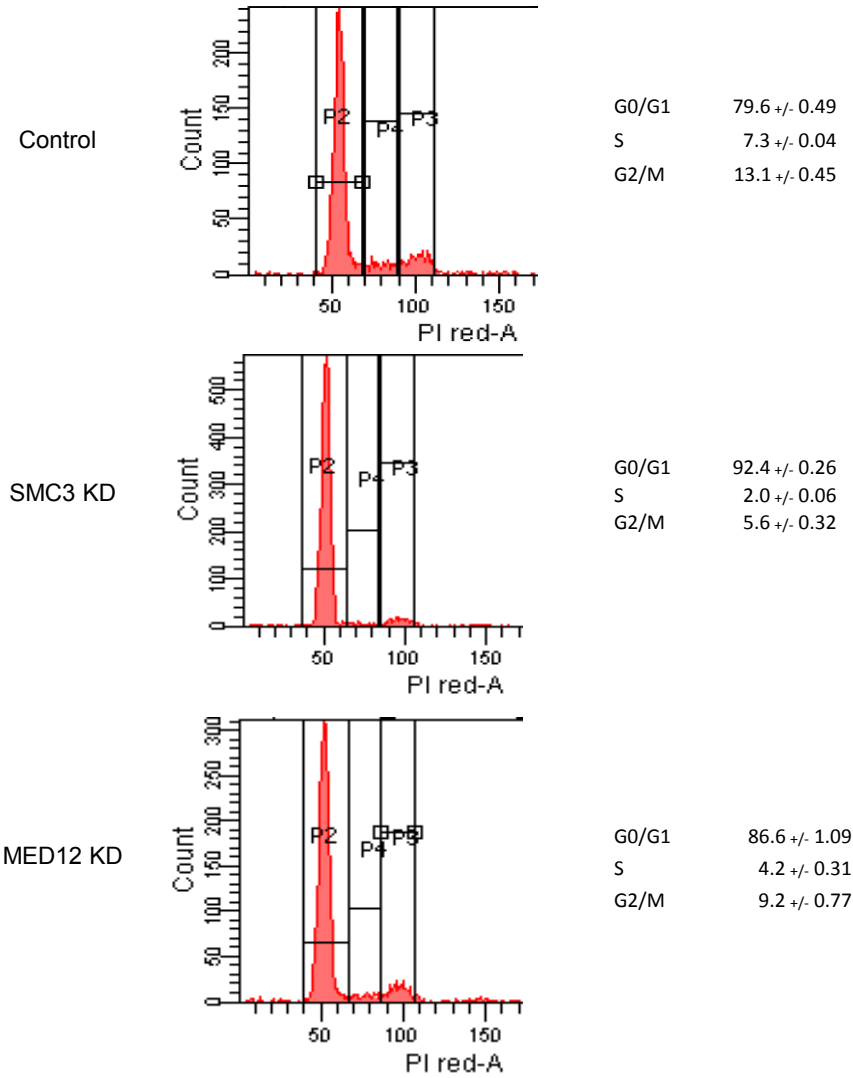

Supplement: Additional file 1 — Figure S1. RAD21 depletion significantly reduces the estrogen-induced expression of ERα target genes. MCF7 cells transfected with control or RAD21 siRNA were grown in normal growth medium for 24 h and then for another 42 h in hormone-depleted medium before treating with 10 nM 17β-Estradiol (E2) for 6 h as indicated. Total mRNA was extracted, reverse-transcribed and analyzed by quantitative real-time PCR (qRT-PCR). (A) The expression levels of the estrogen-regulated genes CXCL12, GREB1, PGR, and PKIB were normalized to 28S ribosomal mRNA, graphed relative to the control sample and expressed as relative mRNA expression; mean values + SD, n = 2. (B) Efficient knockdown of RAD21 and its effects on ESR1 mRNA levels was verified by qRT-PCR in the same samples used in (A). RAD21 and ESR1 were normalized and expressed as in (A). Mean values + SD, n = 2. Figure S2. SMC3 or MED12 knockdown enhance hormone-withdrawal induced G1 cell cycle arrest. MCF7 cells transfected with control, SMC3 or MED12 siRNA were grown in normal growth medium for 24 h and then for another 42 h in hormone-depleted medium before flow cytometric analyses. Shown are representative profiles and the respective quantitation for duplicate samples (± SD). [file 1756-8935-5-13-S1.pdf]
